# Fortification of set yogurt with *Tribute citrus* essential oil: effects on physicochemical properties, multi-spectroscopic features, and microstructure

**DOI:** 10.3389/fnut.2026.1754468

**Published:** 2026-02-18

**Authors:** Kaixiang Jia, Jie Liu, Shengshuang Yu, Jing Wu, Ying Huang, Yule Peng, Wei Wang, Ying Zhao, Yiruo Jin, Peibei Sun, Guoqiang Li

**Affiliations:** 1 School of Food Science and Engineering, Hangzhou Medical College, Hangzhou, Zhejiang, China; 2 School of Biological Science and Food Engineering, Chuzhou University, Chuzhou, China; 3 Zhe Jiang Instution of Tianjin University, Shaoxing, Zhejiang, China; 4 Institute of Advanced Technology, University of Science and Technology of China, Hefei, China

**Keywords:** essential oil, GC-IMS, multi-spectroscopic, set yogurt, *Tribute citrus*

## Abstract

This study aimed to develop an alternative yogurt product with high consumer acceptance by evaluating the effects of *Tribute citrus* essential oil (TCEO) at various concentrations (0–10‰) on its physicochemical properties, multi-spectroscopic characteristics, microstructure, and sensory functionality. Increasing the essential oil (EO) concentration enhanced key parameters of set yogurt: The water-holding capacity increased from 0.64 (control) to 0.98 (10‰) (*p* < 0.05). Rheological behavior was altered, with the crystalline angle (2*θ*) decreasing to approximately 20°. The glass transition temperature also decreased from 76.45 °C to 74.32 °C (*p* < 0.05). A total of 88 volatile organic compound (VOC) fingerprints were identified using headspace gas chromatography-ion mobility spectrometry (HS-GC-IMS), contributing 93.6% to the principal component analysis (PCA). Analysis through scanning electron microscopy (SEM) further highlighted a more uniform and dense microstructure. This research demonstrated the feasibility of fabricating high-quality EO-fortified set yogurt.

## 1 Introduction

Yogurt, well known for its potential benefits as a functional fermented dairy product, has gained widespread consumer acceptance, effectively relieving chronic constipation, protecting the liver to some extent, and lowering blood cholesterol and blood sugar levels (1). Compared to stirred yogurt, set yogurt exhibited a more compact structure, improved cohesiveness, and a longer shelf life (2, 3). These improvements are primarily attributed to the formation of a unique three-dimensional, multi-scale gel network during fermentation, which results from the interaction between casein micelles and whey proteins present in the raw materials (4). This gel structure is characterized by hydrophobic interactions and electrostatic bonding aggregation, encompassing nanostructures, ultramicroscopic systems, and boundary-scale systems, which together contribute to the strength of the multi-scale gels (5–7). With growing consumer interest in nutritional quality and healthy foods, set yogurt incorporated with plant-derived ingredients and essential oils has become particularly appealing due to its distinctive tastes and fragrances. The incorporation of these bioactive ingredients, including phenolic compounds with antioxidant and antibacterial properties, enhances both the taste and health benefits of the yogurt, making it attractive to health-conscious consumers (8, 9).

Essential oil (EO) has been increasingly used as an innovative additive in fermented dairy products such as yogurt, milk drinks, and ice cream due to its diverse functional properties, including antibacterial, antioxidant, anti-inflammatory, and anticancer activities (10, 11). *Tribute citrus* essential oil (TCEO) contains a variety of volatile functional active components, with D-limonene being the most prevalent. Other significant components include *α*-pinene, myrcene, phellandrene, carene, and linalool, which together account for approximately 90% of the essential oil content (12). The addition of EOs to food systems influences both safety and properties. On the one hand, their antibacterial properties can effectively inhibit the growth of common pathogenic bacteria and reduce the need for synthetic preservatives; on the other hand, the complex volatile compounds in EOs add unique flavors, taste, and texture to food products, enhancing the overall sensory experience (13, 14). Additionally, the EO affects the nutritional profile and protein structure of fermented foods, enhancing their functional and sensory characteristics (15, 16). This combination of health benefits and sensory enhancement makes EO an attractive ingredient in functional food development.

The fortification of yogurt with various ingredients, such as carrot juice, rapeseed juice, strawberry juice, and pulp, has garnered significant attention from researchers due to its wide variety of nutrients, including vitamins, minerals, fiber, and bioactive compounds (7, 17). EOs, known for their distinctive flavors and aromas, have been recognized as suitable additives for dairy products and beverages (18–20). As a fortified ingredient, EOs have been homogenized into set yogurt to improve the nutritional content, flavor, and usability (21). However, adding EOs to the set yogurt remains a task that requires extensive research. This is mainly because the high concentrations of EOs can denature casein micelles, leading to whey protein separation, which negatively impacts the texture of the yogurt and causes significant stratification (22, 23). Therefore, further research is necessary to optimize EO concentration and maintain the desired quality of set yogurt.

The incorporation of TCEO into set yogurt has not been extensively explored, particularly regarding its impact on the physicochemical properties of the yogurt. In this study, set yogurt was prepared with TCEO at varying concentrations of 0% (control), 3‰, 6‰, and 10‰. The effects of these varying concentrations on physicochemical properties, dynamic mechanical-thermal properties, morphological characteristics, and consumer-acceptance were investigated. Additionally, an electronic tongue (E-tongue) was employed to further evaluate and discriminate the samples proposed to address discrimination discrepancies and was combined with the principal component analysis (PCA) method to differentiate between samples. The findings of this study aim to provide a theoretical basis for developing a new type of functional set yogurt enriched with TCEO.

## 2 Materials and methods

### 2.1 Materials

The *Tribute citrus* (Wuming, Guangxi, China) was gathered in October 2021. Deionized water was produced using a Milli-Q water purification system (Millipore, Waltham, MA, USA). Milk Powder was sourced from Fonterra Brands New Zealand Ltd. Sugar was purchased from Guangzhou Fuzheng Donghai Food Co. The fermentation strains used in the study (*Streptococcus thermophilus*, *Lactobacillus delbrueckii subsp. bulgaricus*, *Bifidobacterium bifidum,* and *Lactobacillus plantarum*) were supplied by Yili Dairy Products Co.

### 2.2 Preparation of set yogurt

**Figure 1:**
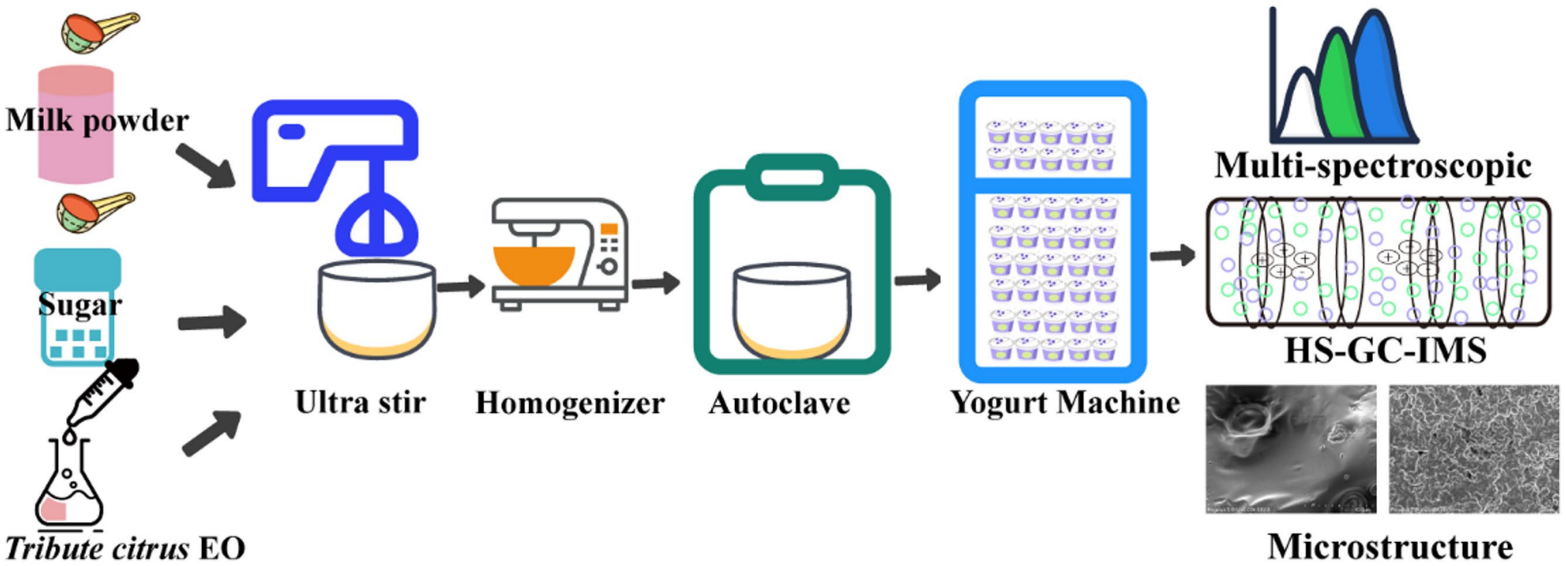
A schematic representation of the preparation of set yogurt incorporated with *Tribute citrus* EO.

The preparation of set yogurt was based on the method of Rong bo Fan et al. (3) with slight modifications. Briefly, 100 mL of distilled water was used to dissolve 12 g of whole milk powder and 6 g of sugar. The mixture was gently stirred and then placed in an ultrasonic cleaner to ensure complete dissolution. Following this, the sonicated mixture was homogenized using a T25 high-speed disperser at 10,000 rpm for 1 min. It was then homogenized in a TAS homogenizer(ATS kind of homogenizer brand) at 600 bar for three cycles. Finally, the mixture was transferred to a 90 °C autoclave for 10 min and then cooled to room temperature. Subsequently, 10.9 ppm (w/w) of yogurt starters were inoculated into a 1 L milk-based solution containing varying concentrations of TCEO (0‰ [control], 3‰, 6‰, and 10‰). The mixture was poured into sterile 200 mL PP plastic cups (8 cm high, 8 cm in diameter) for fermentation. The fermentation was conducted at 42 °C until the yogurt reached a pH of 4.5. After fermentation, the set yogurt samples were stored at 4 °C for 24 h before being promptly evaluated for their physicochemical properties. A schematic representation of the preparation of set yogurt incorporating TCEO is displayed in Figure 1.

### 2.3 Texture characteristics

The texture characteristics of the set yogurt were measured using a TA. The XT Plus texture analyzer (Stable Micro Systems, Surrey, UK) is equipped with a 25 N load cell and a P0.5 acrylic cylinder probe. Hardness and cohesion were measured using the following parameters: test speed of 1.0 mm/s, a compression distance of 20 mm, and a trigger force of 0.1 N.

### 2.4 Physicochemical characterization

#### 2.4.1 Water-holding capacity (WHC)

A 20 g sample of yogurt was centrifuged at 4,000 × g for 10 min at 4 °C. The WHC was measured by weighing the precipitate (designated *m*) after removing the supernatant using Equation 1.


$$\mathrm{WHC}=\frac{m}{20}\times 100.$$ (1)

#### 2.4.2 Measurement of pH and conductivity

The pH and conductivity of the mixture were measured at room temperature using pH meters and conductivity meters, respectively.

### 2.5 Rheological properties

A TA rheometer equipped with a 60 mm cone plate was used to measure the rheological properties of set yogurt under the following conditions: initial gap of 1,000 μm, equilibrated for 2 min before testing.

#### 2.5.1 Steady-state flow test

The flow pattern investigated the effect of shear rate on the viscosity of control and various concentrations of EO-set yogurt, ranging from 0.01 to 1,000 s^−1^. The scanning strain was 0.1%, and the frequency was 0.1 Hz.

#### 2.5.2 Dynamic viscoelasticity test

The storage modulus and loss modulus played an important role in indicating the viscosity and elasticity of the EO-set yogurt. At room temperature, the test mode used was the scanning strain: 0.1%; scanning frequency: 0.1–100 Hz. The fluctuation in energy storage modulus and loss modulus with rotational frequency in the linear viscoelastic zone of the kinematic frequency scan of the EO- set yogurt were analyzed using the following equations.


$$G^{\prime }=K^{\prime }{\left(\omega \right)}^{n^{\prime }}$$ (2)

$$G^{\prime \prime }=K^{\prime \prime }{\left(\omega \right)}^{n^{\prime \prime }}$$ (3)

Where G′ is the storage modulus Equation 2; G′′ is the loss modulus Equation 3; n′ and n′′ are the correlation coefficients of storage modulus and loss modulus; and ω is frequency. Additionally, K′ is correlated with the elastic gel structure in the yogurt sample, and K′ denotes the energy stored and recovered per sinusoidal shear deformation observation at 1 Hz vibration frequency. K′′ is the energy stored and recovered per sinusoidal shear deformation cycle. The viscosity of the yogurt sample is primarily responsible for the energy released as heat, which is intermittently lost.

### 2.6 Fourier transform infrared spectroscopy (FTIR)

FTIR analysis (ATR mode) was performed to investigate the bonds of the yogurt matrix and essential oil in the range of 40–4,000 cm^−1^, with a resolution of 32 (Nicolet iS 5, Thermo Scientific, America).

### 2.7 X-ray diffractometer (XRD)

The X-ray diffractometer (X-Pert PROMPD, Siemens, Germany) was used to measure the diffraction angle (2*θ*) region from 5° to 80° at room temperature.

### 2.8 Differential scanning calorimetry (DSC)

The specimen was dissected and heated at a rate of 10 °C/min before being submitted to a differential scanning calorimetry analyzer in N_2_ circumstances. The temperature was maintained at 200 °C for 5 min, after which it was cooled down to room temperature at a rate of 10 °C/min. Finally, a differential thermal curve was obtained at a heating rate of 10 °C/min to 300 °C.

### 2.9 Thermal analysis

Approximately, a 5 mg set yogurt sample was placed on an aluminum plate and analyzed using a thermogravimetric analyzer (8000, PE, UK). The analysis was conducted from 30 °C to 600 °C at a heating rate of 10 °C/min under an N_2_ flow of 20 mL/min. Furthermore, DTG was used to develop the data available and interaction.

### 2.10 Gas chromatography-ion mobility spectrometry (GC-IMS)

The FlavourSpec^®^ flavor analyzer, namely gas chromatography-ion mobility spectrometry (GC-IMS) technology, was used to investigate volatile compounds in set yogurt samples incorporated with EOs. Three plug-ins and volatile organic compounds (VOCs) were equipped with the device to enable sample analysis from multiple angles; details of test conditions are listed in Supplementary Tables S1, S2.

### 2.11 Scanning electron microscope (SEM)

A particular weight of yogurt samples was pre-frozen at −18 °C for 5 h and then frozen at −80 °C for 24 h. The samples were lyophilized for 48 h. A few lyophilized samples were lightly gold-sprayed, evacuated, and examined using scanning electron microscopy at a 10 kV accelerating voltage.

### 2.12 Electronic tongue measurement

The sensor and the reference electrode of the E-tongue were activated for 24 h before use. Approximately 80 g of EO-set yogurt was verified by an E-tongue equipped with eight flavor sensors, including bitterness, saltiness, astringency, freshness, sour aftertaste, bitter aftertaste, and fresh aftertaste. Each sample was performed in sextuplicate for data analysis.

### 2.13 Statistical analysis

All tests were performed in triplicate (*n* ≥ 3). One-way analysis of variance (ANOVA) was performed, and the treatments were compared through Duncan’s multiple range test at a 5% probability level using SPSS software (version 20.2, SPSS Inc., Chicago, IL). Additionally, Origin Lab software (2018b) was used to plot figures.

## 3 Results and discussion

### 3.1 Texture characteristics of TCEO set yogurt

**Figure 2:**
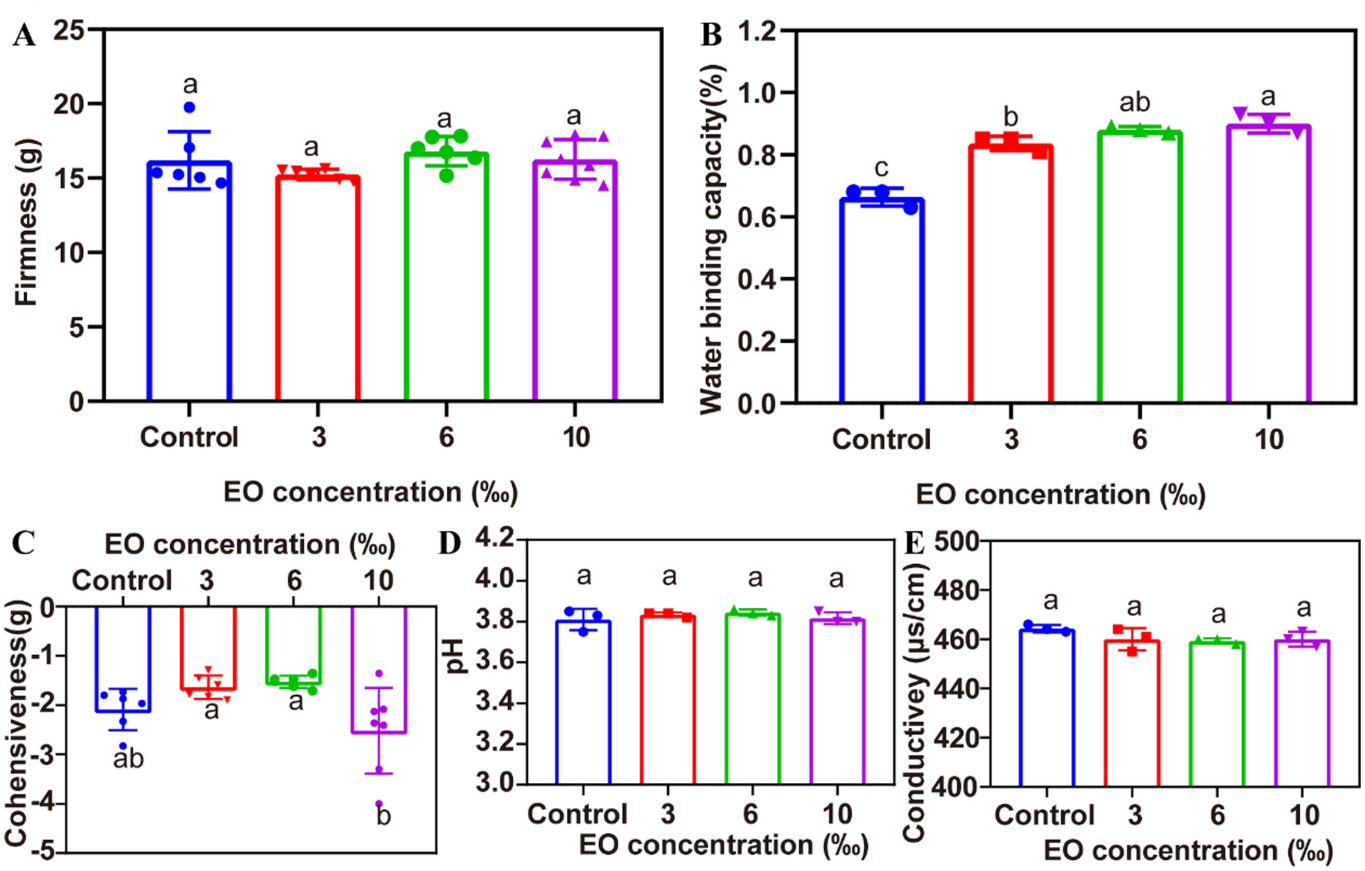
The effect of various concentrations of TCEO on the set yogurt physiochemical properties. **(A)** Firmness, **(B)** water holding capacity, **(C)** cohesiveness, **(D)** pH, **(E)** conductivity.

The effect of EOs on the hardness and adhesion of set yogurt are presented in Figures 2A,C. The hardness of the control sample (without EO) was 15.47 g. However, when EOs were incorporated at various concentrations of 3‰, 6‰, and 10‰, the set yogurt’s hardness altered slightly, with values of 15.24 g, 16.81 g, and 16.32 g, respectively. The slight changes in hardness observed after essential oil (EO) incorporation are likely associated with interactions between EO components and the yogurt protein matrix. Hydrophobic constituents of the EO can interact with milk proteins through hydrophobic interactions, leading to localized modifications in the casein network. Such interactions may result in a more compact or partially reinforced gel structure at specific EO concentrations, thereby influencing hardness (24). Similarly, this trend is consistent with findings by Keshavarzi et al. (25), who focused on adding the ethanol extract and EO from *Ferulago angulate* into set yogurt, resulting in increased hardness, viscosity, and water absorption of the yogurt. Meanwhile, He (26) explored the impact of *Perilla leaf essential oil* (PLEO) on potato yogurt and found that PLEO effectively improved hardness, pH, temperature, and storage duration. The effect of EO on set yogurt adhesion is displayed in Figure 2C. With increasing EO concentrations, there was a substantial (*p* < 0.05) decrease in adhesion, which may be related to the hydrophobic properties of EOs, causing a decrease in water absorption (27). The interaction between the yogurt matrix and the EO likely enhances the water-holding capacity of the yogurt by increasing the availability of hydroxyl groups, which play a critical role in water absorption (28).

### 3.2 Physicochemical properties of TCEO set yogurt

The effects of EO on the physicochemical properties of set yogurt, including pH, water-holding capacity (WHC), and conductivity, are depicted in Figures 2B,D,E, respectively. Compared to the control, a significant increase (*p* < 0.05) in WHC was observed with the increasing concentration of EOs in set yogurt (Figure 2B). This enhancement in WHC aligns with the findings of several previous works, where the formation of a strong three-dimensional multi-scale gel network was found during yogurt fermentation (5–7). The gel structure was formed by casein and whey proteins, which underwent hydrophobic interactions and electrostatic bonding. These interactions give rise to a highly interconnected nanostructure that extends from ultramicroscopic to macroscopic levels, resulting in a gel with superior water retention capabilities (28, 29).

Conversely, the pH and conductivity of the set yogurt remained relatively unchanged across different EO concentrations (pH = 6.5) when compared to the control (*p* < 0.05), as illustrated in Figures 2D,E. This indicates that EO supplementation does not markedly influence the overall acidity or electrical properties of the yogurt, which are essential for maintaining appropriate fermentation dynamics and supporting microbial activity throughout the set yogurt production process. Similar observations have been reported by Hamed et al. (28) who found that the incorporation of essential oils resulted in only minor changes in the pH of yogurt.

### 3.3 Rheological properties

**Figure 3:**
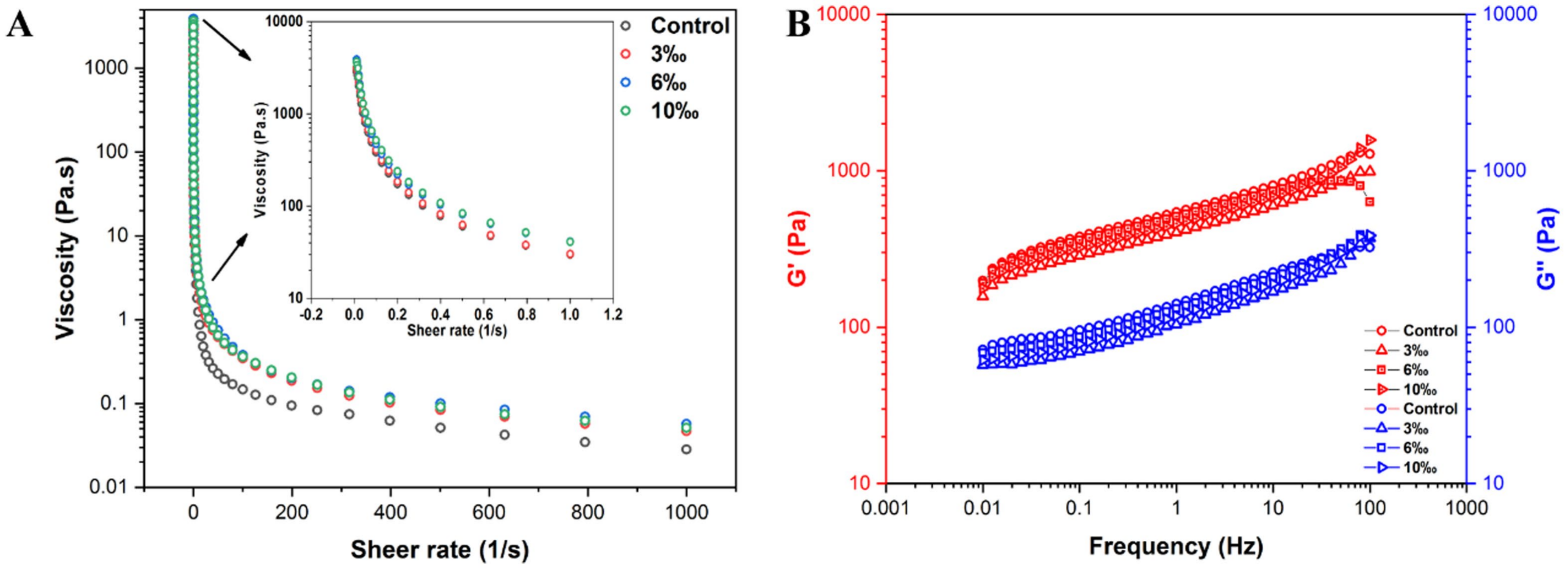
Rheology properties of EO fortified set yogurt: **(A)** steady-flow viscosity, **(B)** storage modulus, and loss modulus.

The rheology flow curves of set yogurt incorporated with various concentrations of EO were measured using a TA rheometer. The flow curves demonstrated a significant non-Newtonian fluid characteristic, as shown in Figure 3A. The non-Newtonian behavior arose due to the rate of recovery under shear stress exceeding the rate of deformation under shear. Specifically, the polymer chains within the yogurt’s fluid matrix disintegrate and rearrange along the flow direction as the shear rate increases beyond the low shear stress threshold (3, 30). Since the rearrangement occurs at a slower rate than the deformation caused by shear forces, the system exhibits shear-thinning properties, consistent with findings from Vukić et al. (31).

According to the inset plot of Figure 3A, noticeable differences in the initial viscosity of yogurt with various concentrations of EO were observed, as reflected by the fluctuations in viscosity at shear rate from 0.01 to 1.0 s^−1^. As the shear rate increased, all samples exhibited pronounced shear-thinning behavior typical of non-Newtonian fluids. This trend may be attributed to the higher EO concentrations, which enhance the viscosity of the yogurt matrix. These observations indicate that EO incorporation influences the viscosity profile of set yogurt, with elevated EO levels contributing to increased apparent viscosity (28, 30). A similar finding was reported by Izadi et al. (32), who demonstrated that enriching yogurt with sterols led to enhanced viscosity and hardness, without significant differences in sensory properties such as texture, flavor, or overall acceptance. In addition to concentration effects, the increase in viscosity may also be attributed to interactions between yogurt milk proteins and EO components. Essential oils contain hydrophobic and phenolic compounds that can interact with casein and whey proteins through hydrophobic interactions and weak non-covalent bonding (4). These interactions may reinforce the protein network, promote gel compactness, and limit protein mobility, thereby enhancing the viscosity and overall structural stability. Similar protein–bioactive compound interactions have been reported to improve the rheological and physical properties of fermented dairy products (33, 34).

The viscoelastic properties of set yogurt supplemented with EO were further evaluated through a frequency scan, as shown in Figure 3B. The storage modulus (G′) was consistently greater than the loss modulus (G′′), indicating a predominantly elastic response, particularly with tan *δ* < 1, which is a characteristic of solid-like materials. This differentiation in viscoelasticity highlights variations in the strength of the yogurt’s network structure. As the shear rate increased, a pronounced non-linear decrease in viscosity was observed, further supporting the shear-thinning behavior of the yogurt. Additionally, no intersection between G′ and G′′ was detected across the frequency scan range of 0.01 to 100 Hz, indicating that the addition of EO enhanced the structural integrity of the yogurt without a transition from solid-like to liquid-like behavior over the tested frequencies. Taken together, these rheological findings demonstrate that EO not only contributes to the shear-thinning behavior of set yogurt but also enhances its viscoelastic network, potentially improving texture attributes and mouthfeel.

### 3.4 FT-IR analysis

**Figure 4:**
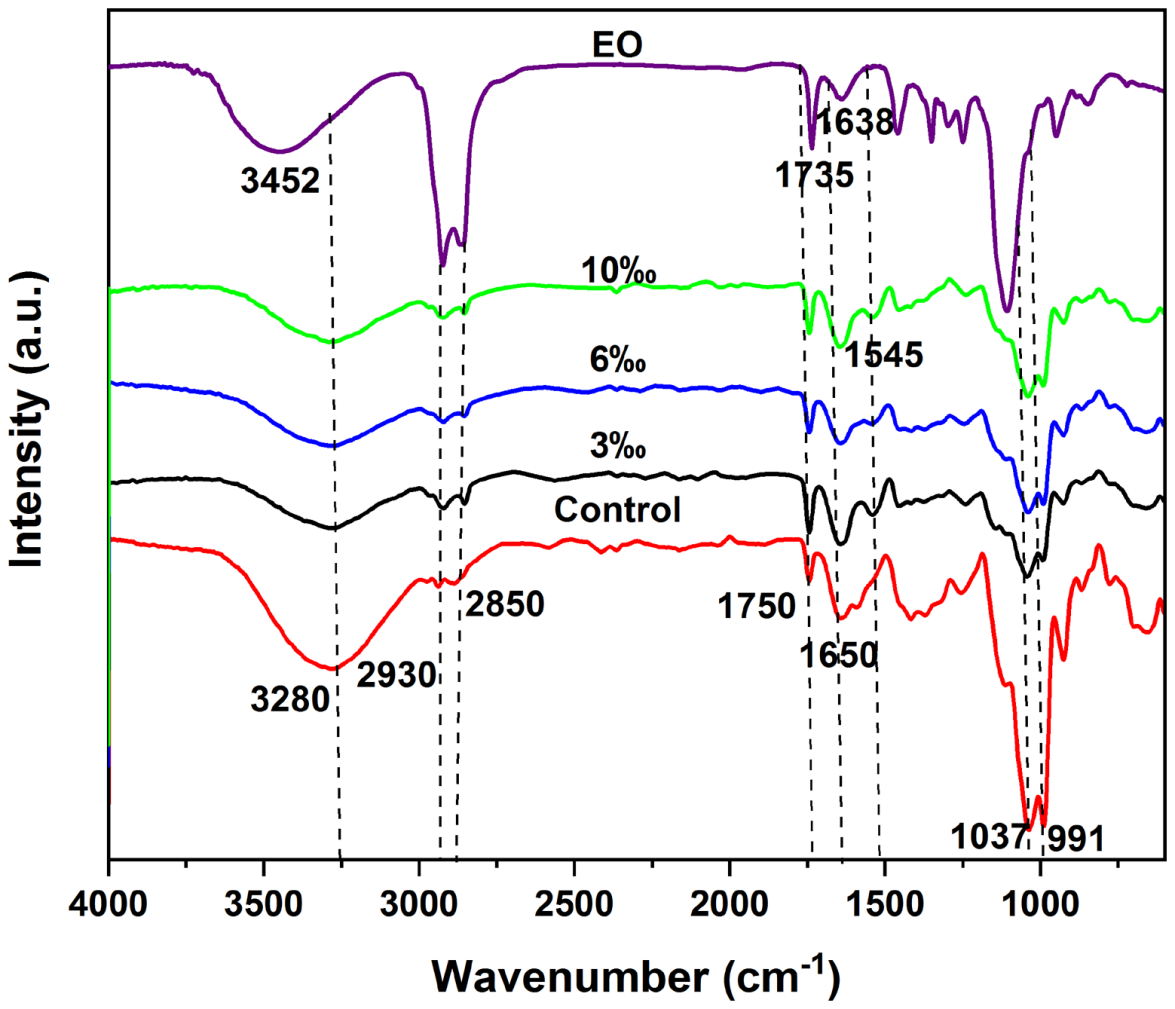
The effect of various of TCEO on the set yogurt by FT-IR.

The FT-IR spectra of set yogurt containing varying concentrations of EO, as illustrated in Figure 4, provided valuable insights into the interaction between EO components and the yogurt matrix. The broad absorbance peak observed in the range of 3,000–3,600 cm^−1^ corresponded to the stretching vibrations of hydroxyl (O-H) groups, consistent with previous findings by Aliyev (35). This broad peak reflects the presence of alcohols within the EO and suggests their involvement in interactions with milk proteins during yogurt formation. The absorption bands at 2,930 cm^−1^ and 2,850 cm^−1^ were assigned to the stretching vibrations of C-H bonds, specifically methylene (-CH₂-) and methyl (-CH₃) groups, which are characteristic of the hydrocarbon components in EO (36). Additionally, peaks at 1,735 cm^−1^ and 1,638 cm^−1^ corresponded to the C=C stretching vibrations, suggesting the presence of unsaturated hydrocarbons such as aromatic compounds within the EOs. Further confirmation of aromatic hydrocarbon content was obtained from peaks at 1,580, 1,500, and 1,450 cm^−1^, each exhibiting variations in intensity associated with different EO concentrations.

The EO-fortified yogurt exhibited a more pronounced peak at 3,280 cm^−1^, extending down to 3,000 cm^−1^, indicating enhanced interactions between the hydroxyl groups of EO and milk proteins. This interaction likely contributed to the reduced intensity of the absorption peak due to hydrogen bonding between EO and protein molecules. A weak peak near 3,000 cm^−1^ was identified as an -NH absorption peak, further suggesting the presence of nitrogen-containing groups within the yogurt matrix. Moreover, a strong absorption peak around 1,750 cm^−1^ corresponded to the presence of carbonyl (C=O) groups. The peak at 1,545 cm^−1^, primarily associated with C=C stretching, confirmed the presence of aromatic or unsaturated structures derived from EO constituents (37).

In the fingerprint region, the absorption band near 1,000 cm^−1^ was attributed to C-O stretching vibrations, indicating that the EO compound may participate in forming new interactions or bonding arrangements with milk proteins. Additionally, the peaks at 1,636 cm^−1^ and 1,037 cm^−1^ corresponded to C=O stretching of carbonyl and carboxyl groups, along with S=O and C-N vibrations, consistent with observations reported by Koperska and Kabir (38, 39). The shifts in the intensity and positioning of some absorption peaks in the FT-IR spectra of EO-enriched yogurt compared to pure EO suggested that the EO components were successfully integrated into the yogurt structure (37). This incorporation likely modified the functional groups within the protein-lipid network, contributing to the enhanced physicochemical properties observed in EO-fortified yogurt.

### 3.5 XRD analysis

**Figure 5:**
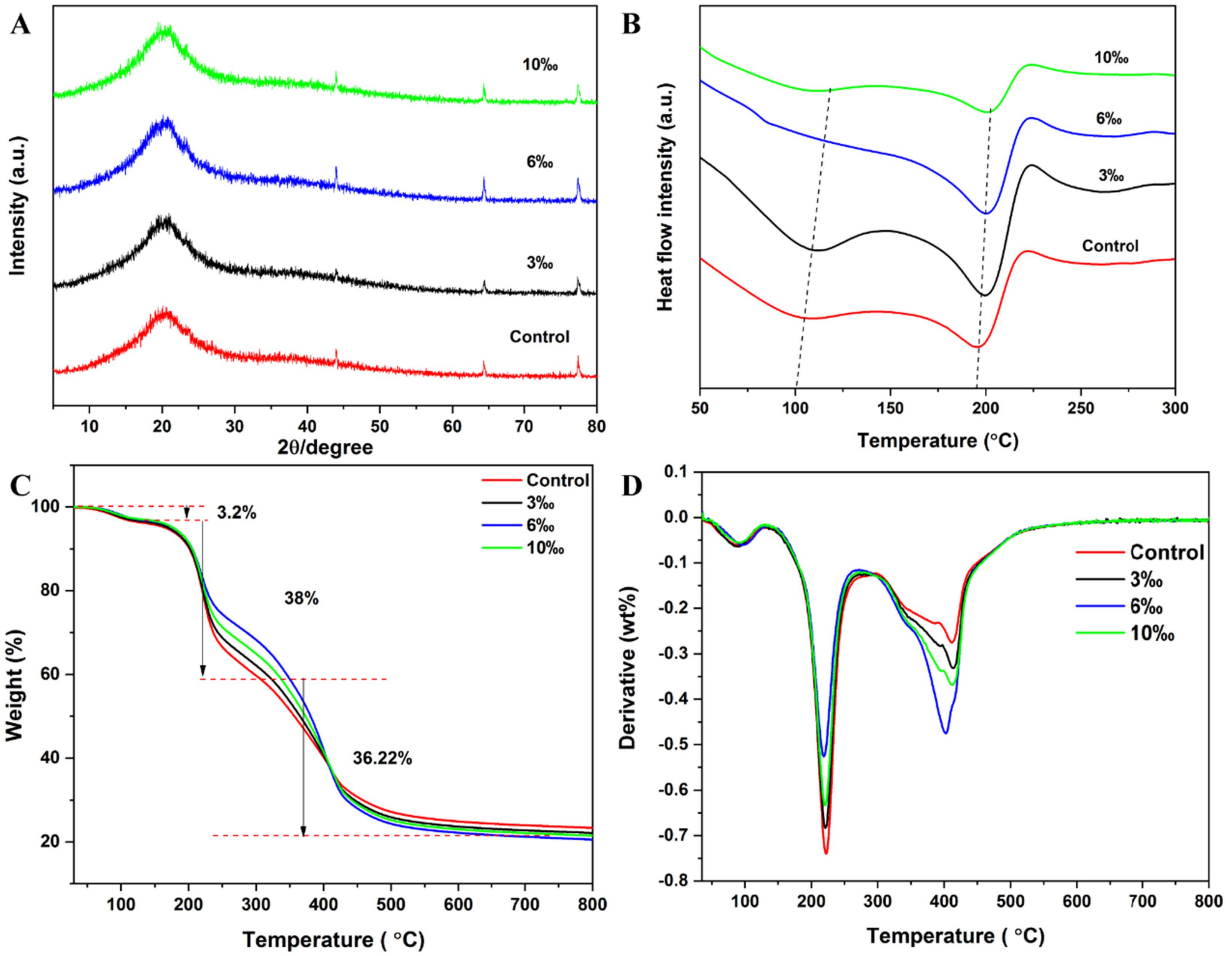
The effect of various of TCEO on set yogurt by XRD, DSC, and TG: **(A)** XRD, **(B)** DSC, **(C)** TG, **(D)** DTG.

The detailed XRD results for the set yogurt are depicted in Figure 5A and Supplementary Table S3. A broad semi-crystalline zone at 2*θ* ≈ 20 ° was identified as the characteristic diffraction peak of yogurt, which progressively scattered and lost strength with the addition of EO. This peak gradually became more diffuse and decreased in intensity with increasing EO concentrations (40). Similar outcomes support the research conducted by El-Sayed (41), who used zinc oxide nanoparticles produced by *Lactobacillus garcia* and added them to set yogurt to study the changes in microbiological, physicochemical, and sensory properties. XRD results revealed that the yogurt system had an effective loading of the zinc oxide nanoparticles.

In general, strong X-ray diffraction occurs along specific crystallographic orientations, and the crystal structure plays a crucial role in determining the intensity and spatial distribution of diffraction patterns. The XRD analysis of yogurt with various EO concentrations revealed potential crystal structures and crystalline forms that could explain the qualitative and rheological features of yogurt (41).

### 3.6 Differential scanning calorimetry analysis

DSC is an important technique for evaluating the curing reaction temperature of polymeric materials and their thermal effects, as shown in Figure 5B. Key thermal properties such as phase transition, thermal effects, crystallization, melting, and glass transition temperature could be observed. Among the above characteristics, glass transition was one of the most crucial elements impacting food quality, processing characteristics, and shelf life. The glass transition of milk powder protein is connected to crystallization, agglomeration, and sedimentation. Additionally, the glass transition temperature may be used to forecast the shelf life and provide the ideal circumstances for food processing and storage to guarantee the security and stability of food storage. The Tg gradually narrowed and fell from 76.45 °C to 74.32 °C. However, there is no glass transition temperature at the concentrations of 3‰ and 6‰. Additionally, the yogurt’s melting points of 173.34 °C, 178.97 °C, 168.02 °C, and 172.15 °C showed that it could keep its glassy condition and was in a reasonably stable state at ambient temperature. The incorporation of TCEO influenced the thermal behavior of yogurt, as reflected by changes in transition temperatures. EO components may interact with milk proteins and lipid fractions, thereby affecting molecular mobility and the structural organization of the yogurt matrix (26).

### 3.7 Thermogravimetric analysis (TG)

TG is a crucial technique for evaluating the heat stability of the materials shown in Figure 5C. The weight of yogurt with different concentrations of EO indicated that the thermal decomposition process could be divided into three events, which were consistent with derivative thermogravimetric analysis (DTG) in Figure 5D. The weight of set yogurt slowly went down at the first event (30–120 °C) and was attributed to the volatility of non-bonded water. Norcino et al. (42) also reported a decrease in weight in pectin film loading with nanoemulsion. The second event (120–270 °C) was related to the vaporization of the volatile essential oils, such as sesquiterpenes. The third event (270–500 °C) was attributed to the boiling point of the solid fraction and the degradation of some terpenoids (43).

In general, the mass of the yogurt containing different concentrations of EO decreased markedly with increasing temperature, following the three distinct decomposition stages in Figures 5C,D. Notably, the sample with 6‰ EO showed a comparatively slow rate with increasing temperature until 270 °C, indicating enhanced thermal stability within the 120–270 °C range, which can be explained by the fact that the proteins in the yogurt were closely surrounded by EO with hydroxyl groups (44). Compared with the control sample, there was a slight weight loss at 270–500 °C in yogurt, which could be attributed to the cross-linking of terpenes with the protein network structure in the yogurt.

### 3.8 Gas chromatography ion mobility spectrometry (GC-IMS)

**Figure 6:**
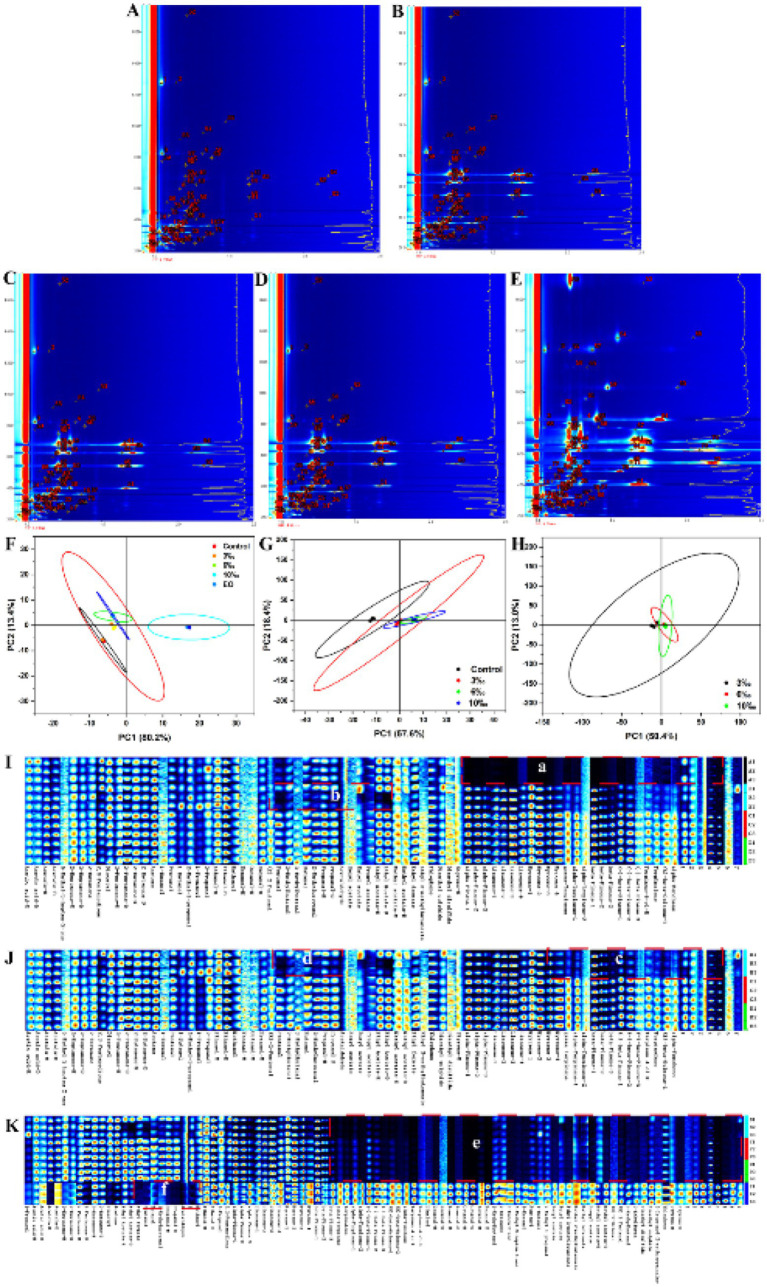
Top view of TCEO set yogurt GC-IMS fingerprint and PCA. **(A)** Top view control, **(B)** top view 3‰ EO set yogurt, **(C)** 6‰ EO set yogurt, **(D)** 10‰ EO set yogurt, **(E)** EO, **(F, G)** PCA (control, EO, and various concentration EO), **(H)** PCA (various concentration EO), **(I–K)** GC-IMS fingerprint (3‰, 6‰, 10‰, EO).

With no specific pretreatment sample solutions required, GC-IMS combines the benefits of gas chromatography’s excellent separation with the high sensitivity of IMS to quickly identify significant quantities or traces of aromatic organics in samples (45). The fingerprint of various concentrations of EO on set yogurt was investigated by HS-GC-IMS in Figures 6A–E. Each signal point in the HS-GC-IMS fingerprint indicated the presence of a VOC, which may produce more than one bright spot (i.e., representing a monomer or dimer). This phenomenon primarily depends on the concentration of the detected VOCs, with the intensity of the relative signal response correlating closely with the concentration of the detected VOCs. Simultaneously, different VOCs had different signal positions on the fingerprint spectrum, while the same substance had corresponding signal intensity at the same position in the fingerprint spectrum. As shown in Table 1, there were 50 VOCs in the control yogurt, 88 VOCs in the EO-fortified yogurt, and 38 VOCs in the EO.

**Table 1:** Analysis of volatile organic compounds of *Tribute citrus* set yogurt by GC-IMS combined with chemometrics.

Count	Compound	CAS#	Formula	MW	RI	Rt (sec)	Dt (RIPrel)	Coefficient type	Comment
1	Acetic acid	C64197	C_2_H_4_O_2_	60.1	1499.9	1270.859	1.05592	+	Monomer
2	Acetic acid	C64197	C_2_H_4_O_2_	60.1	1501.1	1274.167	1.15673	+	Dimer
3	Acetoin	C513860	C_4_H_8_O_2_	88.1	1299.1	821.951	1.06237	+	Monomer
4	Acetoin	C513860	C_4_H_8_O_2_	88.1	1298.6	820.944	1.33002	+	Dimer
5	Pentanol	C71410	C_5_H_12_O	88.1	1264.4	764.507	1.25814	+	
6	2-Heptanone	C110430	C_7_H_14_O	114.2	1196.1	663.728	1.26426	+	Monomer
7	2-Heptanone	C110430	C_7_H_14_O	114.2	1194.6	661.713	1.63285	+	Dimer
8	1-Butanol	C71363	C_4_H_10_O	74.1	1161.7	595.198	1.18014	−	
9	2-Methyl-1-propanol	C78831	C_4_H_10_O	74.1	1107.6	497.32	1.17168	−	
10	2-Hexanone	C591786	C_6_H_12_O	100.2	1100.0	484.965	1.18544	−	
11	Hexanal	C66251	C_6_H_12_O	100.2	1102.8	489.517	1.25701	+	Monomer
12	2,3-Pentanedione	C600146	C_5_H_8_O_2_	100.1	1079.4	455.704	1.22535	+	
13	1-Propanol	C71238	C_3_H_8_O	60.1	1055.4	424.493	1.10837	−	
14	Thiophene	C110021	C_4_H_4_S	84.1	1031.4	395.335	1.03257	+	
15	Diacetyl	C431038	C_4_H_6_O_2_	86.1	1000.1	360.308	1.17372	−	
16	2-Pentanone	C107879	C_5_H_10_O	86.1	1000.5	360.736	1.36877	+	Dimer
17	2-Pentanone	C107879	C_5_H_10_O	86.1	999.7	359.881	1.12111	+	Monomer
18	Ethanol	C64175	C_2_H_6_O	46.1	947.8	319.301	1.04155	−	Monomer
19	Ethanol	C64175	C_2_H_6_O	46.1	947.2	318.874	1.12753	−	Dimer
20	2-Propanol	C67630	C_3_H_8_O	60.1	937.0	311.613	1.08903	+	
21	2-Butanone	C78933	C_4_H_8_O	72.1	920.3	300.079	1.05823	+	Monomer
22	2-Butanone	C78933	C_4_H_8_O	72.1	920.3	300.079	1.24815	+	Dimer
23	Ethyl Acetate	C141786	C_4_H_8_O_2_	88.1	900.2	286.837	1.09801	+	Monomer
24	Ethyl Acetate	C141786	C_4_H_8_O_2_	88.1	900.9	287.265	1.33669	+	Dimer
25	Methanol	C67561	CH_4_O	32.0	912.0	294.526	0.97354	+	
26	Acetone	C67641	C_3_H_6_O	58.1	842.4	251.811	1.11726	−	
27	Propanal	C123386	C_3_H_6_O	58.1	823.2	241.132	1.06336	+	Monomer
28	Propanal	C123386	C_3_H_6_O	58.1	823.2	241.132	1.14934	+	Dimer
29	Methyl acetate	C79209	C_3_H_6_O_2_	74.1	852.1	257.364	1.0454	+	Monomer
30	Ethyl formate	C109944	C_3_H_6_O_2_	74.1	838.6	249.675	1.20195	+	
31	2-Methylpropanal	C78842	C_4_H_8_O	72.1	835.6	247.966	1.28023	+	
32	Dimethyl sulphide	C75183	C_2_H_6_S	62.1	801.4	229.598	0.95814	−	
33	Acetaldehyde	C75070	C_2_H_4_O	44.1	776.8	217.211	0.97995	+	
34	Butanal	C123728	C_4_H_8_O	72.1	892.9	282.139	1.11983	+	
35	alpha-Pinene	C80568	C_10_H_16_	136.2	1035.4	400.034	1.21478	−	1
36	alpha-Pinene	C80568	C_10_H_16_	136.2	1036.1	400.888	1.28793	−	2
37	Pentanal	C110623	C_5_H_10_O	86.1	1004.1	364.58	1.42138	+	
38	2-Methylbutanal	C96173	C_5_H_10_O	86.1	929.6	306.487	1.15832	+	
39	Limonene	C138863	C_10_H_16_	136.2	1204.5	675.349	1.21636	−	1
40	Limonene	C138863	C_10_H_16_	136.2	1205.1	676.294	1.2941	−	2
41	Limonene	C138863	C_10_H_16_	136.2	1205.8	677.24	1.6603	−	3
42	Myrcene	C123353	C_10_H_16_	136.2	1175.6	623.357	1.21432	−	1
43	Myrcene	C123353	C_10_H_16_	136.2	1175.6	623.357	1.28796	−	2
44	Myrcene	C123353	C_10_H_16_	136.2	1176.6	625.247	1.63984	−	3
45	gamma-Terpinene	C99854	C_10_H_16_	136.2	1251.5	744.357	1.21841	+	
46	alpha-Terpinene	C99865	C_10_H_16_	136.2	1187.3	647.935	1.21432	+	1
47	beta-Pinene	C127913	C_10_H_16_	136.2	1134.1	543.005	1.21432	−	1
48	beta-Pinene	C127913	C_10_H_16_	136.2	1135.1	544.896	1.63984	+	2
49	(−)-beta-Pinene	C18172673	C_10_H_16_	136.2	1120.1	518.431	1.21586	+	1
50	(−)-beta-Pinene	C18172673	C_10_H_16_	136.2	1120.4	518.989	1.30201	+	2
51	alpha-Pinene	C80568	C_10_H_16_	136.2	1035.6	400.232	1.66449	+	3
52	Terpinen-4-ol	C562743	C_10_H_18_O	154.3	1635.0	1703.626	1.22649	+	Monomer
53	Terpinen-4-ol	C562743	C_10_H_18_O	154.3	1636.2	1708.104	1.72065	+	Dimer
54	Decanal	C112312	C_10_H_20_O	156.3	1527.8	1349.917	1.54799	−	Monomer
55	Decanal	C112312	C_10_H_20_O	156.3	1528.5	1352.155	2.05407	−	Dimer
56	Linalool	C78706	C_10_H_18_O	154.3	1499.9	1270.86	1.22975	−	
57	3-Nonen-2-one	C14309570	C_9_H_16_O	140.2	1500.5	1272.435	1.37806	−	
58	3-Isopropyl-2-methoxypyrazine	C25773404	C_8_H_12_N_2_O	152.2	1460.4	1166.399	1.25558	−	
59	Nonanal	C124196	C_9_H_18_O	142.2	1401.3	1025.904	1.48288	−	Monomer
60	Nonanal	C124196	C_9_H_18_O	142.2	1403.0	1029.771	1.94942	−	Dimer
61	1-Hexanol	C111273	C_6_H_14_O	102.2	1367.7	953.724	1.33534	−	
62	6-Methyl-5-hepten-2-one	C110930	C_8_H_14_O	126.2	1347.9	913.766	1.18182	−	
63	Octanal	C124130	C_8_H_16_O	128.2	1298.5	820.782	1.40548	+	Monomer
64	Octanal	C124130	C_8_H_16_O	128.2	1298.5	820.782	1.82832	+	Dimer
65	Terpinolene	C586629	C_10_H_16_	136.2	1285.5	798.737	1.22374	+	
66	(E)-beta-Ocimene	C3779611	C_10_H_16_	136.2	1261.3	759.736	1.21835	+	1
67	(E)-beta-Ocimene	C3779611	C_10_H_16_	136.2	1260.8	758.888	1.66638	−	2
68	Styrene	C100425	C_8_H_8_	104.2	1267.8	769.91	1.06001	−	Monomer
69	Styrene	C100425	C_8_H_8_	104.2	1268.8	771.606	1.41987	−	Dimer
70	Hexyl acetate	C142927	C_8_H_16_O_2_	144.2	1282.4	793.65	1.39288	+	
71	(−)-beta-Pinene	C18172673	C_10_H_16_	136.2	1121.4	520.638	1.64299	+	3
72	Myrcene	C123353	C_10_H_16_	136.2	1177.2	626.621	2.09462	+	4
73	beta-Pinene	C127913	C_10_H_16_	136.2	1134.3	543.531	2.18099	−	3
74	Limonene	C138863	C_10_H_16_	136.2	1206.0	677.493	2.16659	+	4
75	alpha-Terpinene	C99865	C_10_H_16_	136.2	1187.6	648.652	1.71636	−	2
76	Hexanal	C66251	C_6_H_12_O	100.2	1102.8	489.534	1.56265	+	Dimer
77	(E)-2-Pentenal	C1576870	C_5_H_8_O	84.1	1115.0	509.789	1.09492	−	
78	Butyl acetate	C123864	C_6_H_12_O_2_	116.2	1090.5	470.967	1.24152	−	
79	alpha-Fenchene	C471841	C_10_H_16_	136.2	1055.4	424.417	1.21443	+	
80	Ethyl 3-methylbutanoate	C108645	C_7_H_14_O_2_	130.2	1067.0	439.374	1.25078	+	
81	Methyl 2-methylbutanoate	C868575	C_6_H_12_O_2_	116.2	1014.5	375.934	1.19625	−	
82	Propyl acetate	C109604	C_5_H_10_O_2_	102.1	989.5	350.772	1.15724	+	
83	Methyl acetate	C79209	C_3_H_6_O_2_	74.1	850.8	256.619	1.19333	−	Dimer
84	Dimethyl disulfide	C624920	C_2_H_6_S_2_	94.2	1086.7	465.707	1.12811	+	
85	Methyl butanoate	C623427	C_5_H_10_O_2_	102.1	1017.4	379.255	1.14938	−	
86	3-Methylbutanal	C590863	C_5_H_10_O	86.1	932.2	308.232	1.40093	+	
87	2-Ethylfuran	C3208160	C_6_H_8_O	96.1	955.6	324.943	1.30244	+	
88	(E)-2-Octenal	C2548870	C_8_H_14_O	126.2	1458.1	1160.642	1.34403	−	

To further evaluate the effect of EOs on yogurt, PCA was performed on different sample groups, as shown in Figures 6F–I. The contributions of PC1 (80.2%), PC2 (13.4%), and a total of 93.6% indicated that the PCA model effectively captured the major information represented in the GC-IMS fingerprints (Figure 6F). In the PCA plot (Figure 6F), the control, EO-set yogurt, and EO showed obvious regional distribution characteristics. The EO samples were far from the other samples, whereas the control and EO set yogurt stayed on the same side. Further relationships between yogurt and EO-fortified yogurt were studied in Figures 6G,H. An obvious regional distribution was observed, dividing the samples into two major clusters (Figure 6G), while the EO-fortified set yogurt samples were grouped in one cluster.

The differential volatile organic compounds identified are highlighted by red boxes labeled a–f in panels I, J, and K of Figure 6, respectively. Among these compounds, a region lacking compounds in the control samples, including 2-propanol, and EO characteristic volatile compounds. Compared to the 6‰ and 10‰ concentrations, the 3‰ EO-fortified set yogurt was slightly lower in the concentration of yogurt flavor compounds.

In dairy products, ketones are typically generated through the metabolic activities of lactic acid bacteria or the oxidation and degradation of unsaturated fatty acids or amino acids in yogurt, of which relatively high levels of 2-butanones in turn have a strong creamy and caramel aroma (46). Esters are another important class of flavor compounds in yogurt, and the combination of fatty acid hydrolysis and microbial human metabolism generates esters that have a low flavor threshold and a large impact on yogurt flavor formation. Ethyl esters in yogurt impart a fruity-floral flavor that reduces the bitterness and fatty acid aftertaste produced by amino acids in yogurt (47). Conversely, aldehyde sources are characteristic flavor metabolites produced by live bacteria during the fermentation of low-fat yogurt, where acetyl compounds impart a refreshing aromatic taste (48).

In general, the response signal of volatile organic compounds was enhanced in 1-propanol, acetic acid, ethyl coupling, 2-heptanone, 2-pentanone, 2-butanone, diacetyl, acetone, ethyl acetate, ethyl formate, glutaraldehyde, butyraldehyde, 2-methyl propionaldehyde, propionaldehyde, and acetaldehyde in the set yogurt compared to the EO. The response values of terpenoids were enhanced in the EO-fortified set yogurt. The enhanced response signals of ketones, aldehydes, and esters such as diacetyl, ethyl coupling, acetaldehyde, and 2-butanone at the same time imparted the yogurt with a special fruit flavor and refreshing aromatic scent.

### 3.9 Scanning electron microscopy (SEM)

**Figure 7:**
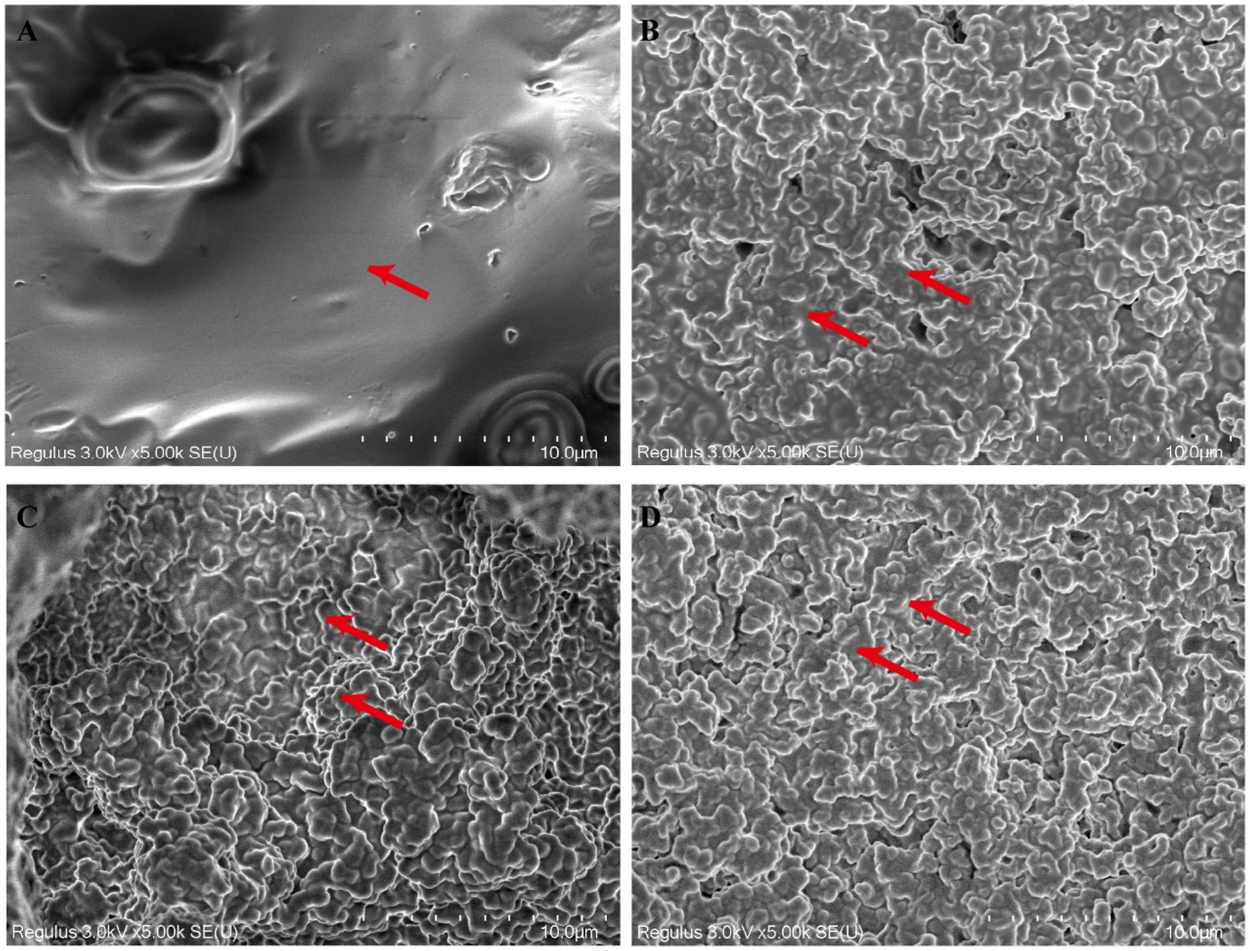
Microstructure of EO fortified set yogurt. **(A)** Control, **(B)** 3‰EO, **(C)** 6‰EO, **(D)** 10‰EO.

The microstructure of set yogurt is crucial in determining its gel strength and overall quality, such as WHC and shelf life. The microstructure of the control and EO-fortified set yogurts observed by SEM is shown in Figure 7 (red arrow). As illustrated in Figure 7A, the microstructure of control samples exhibited non-uniform connections of aggregated protein clusters and appeared as localized aggregated particles, resulting in poor gelation of yogurt, which was not conducive to maintaining high water-holding capacity and long shelf life. The terpenoids, alkenes, and alcohols in the essential oil are conducive to the increase of gel strength of set yogurt with increasing EO concentration, as shown in Figures 7B–D, respectively. The EO-fortified set yogurt displayed a much denser and more uniform protein network. This improvement in the microstructure is linked to the presence of terpenoids, alkenes, and alcohols in the EO, which contributed to increased gel strength. These compounds, particularly aldehydes and esters in the EO, reduce the pH of the yogurt mixture, triggering critical structural changes in the casein. The reduction in pH upon EO addition leads to a significant change in the behavior of casein, as described in prior research (24, 42, 49). When the pH falls below a critical threshold, the *κ*-casein (*κ*-CN) “hair-like” layer surrounding the micelles loses its negative charge and collapses onto the micelle surface. This collapse eliminates the spatial repulsion that normally keeps micelles dispersed, leading to micelle aggregation. As EO concentration increases, the aggregation of protein intensifies, forming a more compact and cohesive three-dimensional network. This denser network structure, visualized in the SEM images, indicates that EO promotes protein denaturation and aggregation, thereby strengthening the yogurt gel. Therefore, SEM analysis of the microstructure clearly demonstrates that EO fortification yields a denser, more uniform protein matrix, thereby enhancing the yogurt gel strength. This improved microstructure not only supports a higher water-holding capacity but also contributes to longer shelf life and improved overall yogurt quality.

### 3.10 Electronic tongue analysis (E-tongue)

**Figure 8:**
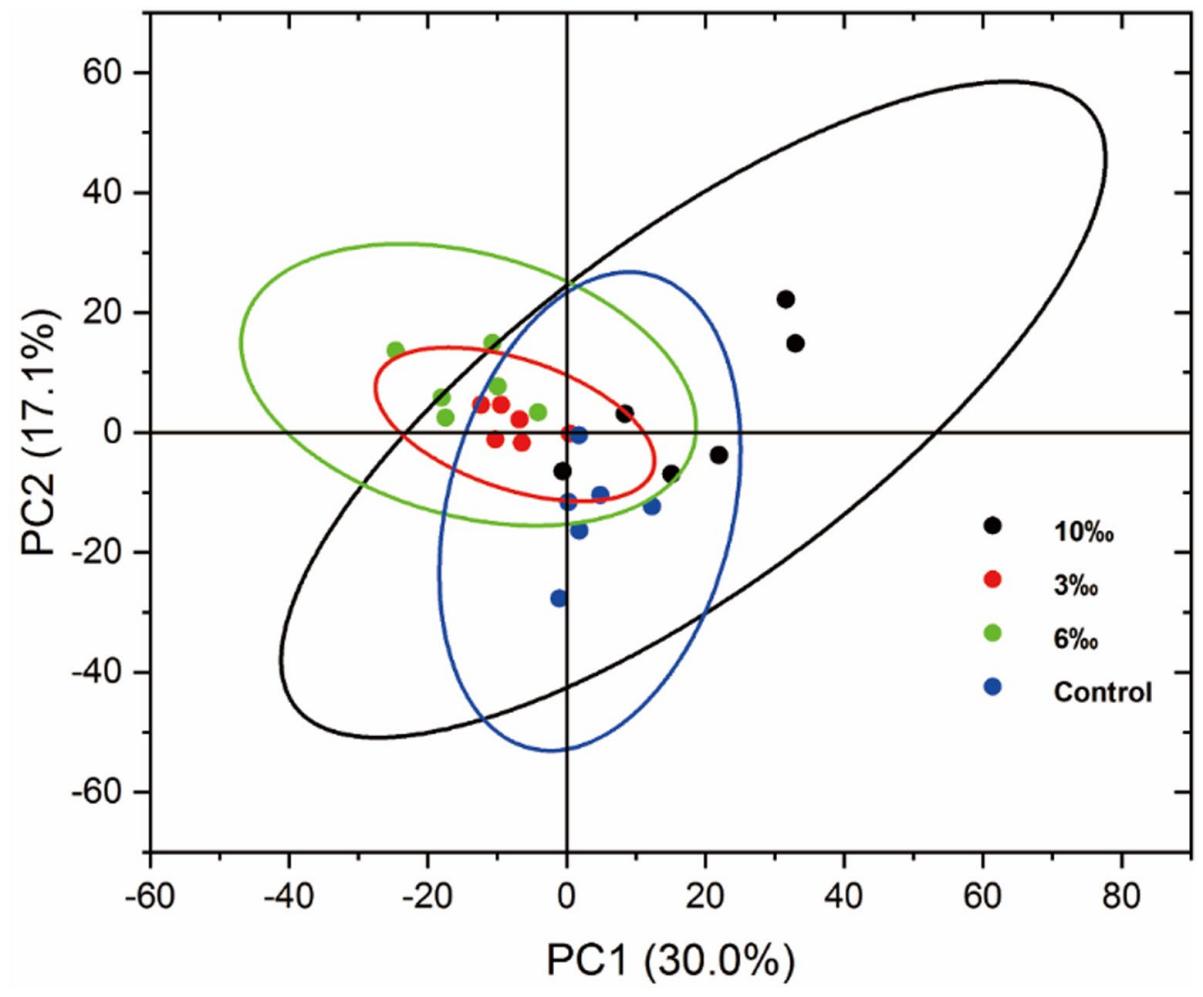
PCA of set yogurt by E-tongue analysis.

The E-tongue was used to analyze and distinguish the sensory profiles of set yogurts fortified with different EO concentrations by detecting five primary human taste sensations (sour, bitter, salty, fresh, and astringent) and three related aftertastes. As shown in Figure 8A, the results of the E-tongue analysis of samples were obtained in the form of a PCA plot. The first two principal components (PC1 30.0% and PC2 17.1%) were found and accounted for approximately 47.1% of the total variance and could well reflect the samples’ flavor information, indicating that PC1 contained the highest amount of information related to sensory components, allowing for an initial grouping of the samples based on taste characteristics. The E-tongue had potentially been used to preliminarily discriminate among flavors via sensor-response analysis. In the PCA diagram, the samples showed obvious regional distribution characteristics; the 3‰ and 6‰ EO-fortified set yogurts were different from the control and the 10‰ EO group in terms of sensory components. The reason was that bioactive compounds in EOs, such as limonene and terpenoids, may enhance flavor complexity and balance by contributing mild bitterness, aromatic notes, and taste-modulating effects. These compounds can interact with taste receptors and the yogurt matrix, leading to distinct sensor responses captured by the E-tongue (46). The 3‰ and 6‰ EO set yogurts showed similar sensory results and clustered to one side, while the sensory results of the control and 10‰ EO set yogurts were cross-linked similarly. It is possible that the alkenes in the yogurt, which contribute to a pungent sour taste were converted into a milder lactic acid by the action of lactic acid bacteria (21).

## 4 Conclusion

In this study, set yogurt fortified with various concentrations of TCEO was evaluated to determine its effects on physicochemical properties, multispectroscopic features, morphological structure, and sensory properties. The EO-fortified set yogurt significantly enhanced water-holding capacity and improved rheology properties, including viscosity and viscoelasticity. TCEO was uniformly dispersed within the yogurt matrix and contributed to increased crystallinity and a higher glass transition temperature, both of which are advantageous for extending shelf life. HS-GC-IMS analysis identified 88 VOCs with a total contribution of 93.6% by PCA, demonstrating a clear differentiation among samples. Furthermore, TCEO improved the structural uniformity of the yogurt, intensified desirable microstructural features, and enhanced aroma and flavor attributes. Furthermore, the effect of TCEO on the storage period should be considered in future studies.

## Data Availability

The raw data supporting the conclusions of this article will be made available by the authors, without undue reservation.
