# Supplementary material for: Fortification of set yogurt with Tribute citrus essential oil: effects on physicochemical properties, multi-spectroscopic features, and microstructure
Source: Front Nutr. 2026 Feb 18;13:1754468. doi: 10.3389/fnut.2026.1754468 (PMC12956710; doi:10.3389/fnut.2026.1754468)
Supplement: Supplementary file 1 [file Table_1.DOCX]

**Fortification of set yogurt with *Tribute citrus* essential oil: Effects on physicochemical properties, multi-spectroscopic, and microstructure**

**Kaixiang Jia^a,†^, Jie Liu^b,†^, Shengshuang Yu^c,†^, Jing Wu^d^, Ying Huang^a^, Yule Peng^a^, Wei Wang^a^, Ying Zhao^a^, Yinuo Jin^a^, Peibei Sun^a^, Guoqiang Li^a*^**

^a^ School of Food Science and Engineering, Hangzhou Medical College, Hangzhou, Zhejiang 311300, China.

^b^ School of Biological Science and Food Engineering, Chuzhou University, Chuzhou, 239000,China

^c^ Zhe Jiang Instution of Tianjin University, Shaoxing, Zhejiang 312000, China.

^d^ Institute of Advanced Technology, University of Science and Technology of China, Hefei 230094, China.

*** Corresponding author:**

*Guoqiang Li

**[guoqli@hmc.edu.cn](mailto:shij136@hmc.edu.cn)**

**+86 571 87292623**

**Keywords:** ***Tribute citrus*; essential oil; set yogurt; multi-spectroscopic**

**^†^These authors contributed equally to this work**

Table S1 Analyze conditions

| GC-IMS unit | |
| --- | --- |
| Time | 30 min |
| chromatographic column | wax，30m，ID:0.53mm，film thickness: 1μm (RESTEK Co. USA) |
| Column temperature | 60 °C |
| Drift gas | N_2_ |
| IMS Temperature | 45 °C |
| Headspace injection unit | |
| Injection volume | 200 ul |
| Incubation time | 10 min |
| Incubation temperature | 45 °C |
| Injection needle temperature | 60 °C |
| Incubation speed | 500 rpm |

Table S2 Gas chromatographic conditions

| Time（min：sec） | E1（Drift gas） | E2（Carrier gas） | Recording |
| --- | --- | --- | --- |
| 00:00 | 150mL/min | 2 ml/min | rec |
| 02:00 | 150mL/min | 2 ml/min | - |
| 10:00 | 150mL/min | 10 ml/min | - |
| 20:00 | 150mL/min | 100 ml/min | - |
| 30:00 | 150mL/min | 100 ml/min | stop |

Table S3 The effect of various ***Tribute citrus*** EO set yoghurt on XRD degree of crystalline

| Sample | Crystalline |
| --- | --- |
| Control | 68.24%b |
| 3‰ | 95.77%a |
| 6‰ | 94.60%a |
| 10‰ | 94.37%a |
